# Functional Characterization of Two Adjacent Oxygenase Genes, *SsOxy1* and *SsOxy2*, Reveals Their Critical Roles in Sclerotial Development, Stress Tolerance, and Pathogenicity of *Sclerotinia sclerotiorum*

**DOI:** 10.3390/jof12090688

**Published:** 2026-09-13

**Authors:** Ruiwen Liu, Cheng Zhu, Zhi Li, Liang Li, Shijie Yu, Bo Song, Yu Xu, Zhi Zhao, Taocui Huang, Yang Yu

**Affiliations:** 1 Chongqing Academy of Agricultural Sciences, Chongqing 401329, China; 18235448445@163.com (R.L.);; 2 College of Plant Protection, Southwest University, Chongqing 400715, China

**Keywords:** *Sclerotinia sclerotiorum*, oxygenase, targeted gene knockout, pathogenicity, infection cushion, metabolomics

## Abstract

*Sclerotinia sclerotiorum* is a notoriously destructive, broad-host-range necrotrophic fungus responsible for devastating crop losses worldwide. Although genes encoding oxygenases are ubiquitous in fungal genomes, and the encoded enzymes play instrumental roles in driving diverse biochemical cascades, their specific functional repertoire in *S. sclerotiorum* pathogenesis remains poorly defined. Here, we characterize a contiguous pair of early-infection-induced oxygenase genes: *SsOxy1* (encoding a 2-oxoglutarate-dependent oxygenase) and *SsOxy2* (encoding a non-heme iron-dependent oxygenase). Targeted gene disruption demonstrated distinct developmental roles: Δ*SsOxy1* mutants exhibited compromised vegetative growth (colony diameter reduced by approximately 18% to 27% at 24 h) and significantly diminished sclerotial biomass (ranging from approximately 8% to 28% reduction), whereas the absence of *SsOxy2* selectively uncoupled the spatial regulation of sclerotial biogenesis without restricting radial growth. Importantly, both mutants displayed a severe decline in virulence across multiple hosts, with lesion diameters reduced by approximately 30% to 67% in Δ*SsOxy1* and 62% to 66% in Δ*SsOxy2* (*p* < 0.01).This attenuation was fundamentally linked to their inability to elaborate mature infection cushions, compounded by hypersensitivity to exogenous oxidative (H_2_O_2_) and hyperosmotic (NaCl) stresses. Untargeted metabolomics uncovered a profound metabolic reprogramming shared by both mutants, characterized by severe depletion of purine metabolism intermediates, including hypoxanthine (4.6–4.9-fold), xanthine (6.1–8.1-fold), and guanine (2.5–3.7-fold), alongside a disrupted glutathione redox pool (elevated GSSG: 3.0-fold in Δ*SsOxy1* and 5.7-fold in Δ*SsOxy2*). Genomic colocalization combined with these metabolomic shifts suggests that *SsOxy1* and *SsOxy2* may function in a coordinated manner within a putative secondary metabolite biosynthetic gene cluster, although direct evidence for co-transcription and a shared product is currently lacking. Our findings provide novel mechanistic insights into how these paired oxygenases govern redox homeostasis, energy allocation, and the critical morphological transitions required for successful host colonization in *S. sclerotiorum*.

## 1. Introduction

*Sclerotinia sclerotiorum* (Lib.) de Bary represents a paradigmatic necrotrophic plant pathogen. With an exceptionally broad host range encompassing hundreds of dicotyledonous species—most notably agriculturally vital Brassica crops and soybeans—it is a perennial threat to global food security [1,2,3,4,5,6,7]. Current disease management strategies are highly dependent on chemical interventions, yet the intensive application of azoxystrobin, carbendazim, and succinate dehydrogenase inhibitors has inevitably selected for multi-drug-resistant field populations [8,9,10]. The epidemiological resilience of this pathogen is largely underpinned by its capacity to differentiate into sclerotia. These highly melanized, resilient survival structures can persist in the soil profile for years, effectively acting as the primary inoculum for recurrent disease cycles [11,12].

Successful host colonization by *S. sclerotiorum* is initiated by the rapid differentiation of vegetative hyphae into compound appressoria, commonly termed infection cushions. These multicellular structures are indispensable for exerting mechanical pressure against the plant cuticle and subsequently deploying an arsenal of virulence factors [13]. Classically, pathogenesis has been attributed to a bipartite offensive strategy: the massive secretion of cell wall-degrading enzymes and the generation of oxalic acid. Oxalic acid facilitates infection by acidifying the local microenvironment, sequestering calcium, and crucially, dampening the host’s early oxidative burst [14,15]. Furthermore, contemporary transcriptomic and functional studies have expanded this paradigm to include a suite of secreted effector proteins, such as SsSSVP1 and SsCP1, which translocate into plant cells to subvert host immunity [16,17,18,19].

Beyond degradative enzymes and effectors, the dynamic fungal secondary metabolome is increasingly recognized as a vital arbiter of host–pathogen interactions [20]. Fungal secondary metabolites—ranging from siderophores and melanins to specialized phytotoxins—govern ecological fitness, morphological transitions, and pathogenic success [21,22,23,24]. The genetic architecture underpinning secondary metabolite biosynthesis is typically modular, organized into contiguous chromosomal biosynthetic gene clusters [25]. Within these clusters, the nascent scaffolds assembled by core synthases invariably require extensive structural modification to attain biological activity. Oxygenases frequently act as predominant tailoring enzymes mediating this maturation process [26,27]. For example, in the biosynthesis of ergot alkaloids and aflatoxin, non-heme iron- and 2-oxoglutarate-dependent oxygenases catalyze key hydroxylation and epoxidation steps that diversify the toxin scaffolds [28,29]. Despite their evolutionary conservation, the regulatory and pathogenic contributions of specific oxygenases in *S. sclerotiorum* remain a distinct knowledge gap. Specifically, no study has systematically investigated whether oxygenase-encoding genes adjacent in the genome play coordinated roles in infection structure development and stress adaptation.

In the present study, we focused on two adjacent, putative oxygenase genes (*SsOxy1* and *SsOxy2*) that are acutely upregulated during the onset of host infection. By generating targeted deletion mutants, we conducted a systematic evaluation of their contributions to vegetative growth, sclerotial ontogeny, stress adaptation, and full virulence. To further elucidate the biochemical basis of the observed mutant phenotypes, we integrated untargeted metabolomic profiling. This combined genetic and multi-omics approach sheds new light on the pleiotropic functions of paired oxygenases in the lifecycle and pathogenic arsenal of *S. sclerotiorum*.

## 2. Materials and Methods

### 2.1. Fungal Strains and Culture Conditions

The wild-type *S. sclerotiorum* strain 1980 (preserved at the College of Plant Protection, Southwest University) and its derivative transformants were routinely cultured on potato dextrose agar (PDA, BD Difco, Franklin Lakes, NJ, USA) at 20 °C in darkness, as sclerotial formation is optimal under dark conditions. For mycelial growth rate assays, uniform mycelial plugs (5 mm in diameter) were inoculated centrally onto PDA plates, and colony diameters were recorded at 24 h intervals. Pathogenicity assays were conducted using detached leaves of oilseed rape (*Brassica napus* cv. ‘Zhongshuang 11’) and intact *Nicotiana benthamiana* plants, both cultivated in a greenhouse maintained at 25 °C under a 16 h light/8 h dark photoperiod.

### 2.2. Gene Structure Analysis and Expression Profiling

Protein domain architectures for SsOxy1 and SsOxy2 were annotated using the NCBI Conserved Domain Database (CDD, https://www.ncbi.nlm.nih.gov/Structure/cdd (accessed on 15 March 2020)). Sequence homology searches were executed via BlastP (e-value cutoff 1 × 10^−5^, coverage ≥ 50%). Phylogenetic relationships were inferred using the neighbor-joining method in MEGA 7.0 (1000 bootstrap replicates). To monitor infection-phase expression dynamics, mycelial samples were harvested from inoculated tobacco leaves at 0, 4, 8, 12, and 24 h post-inoculation (hpi). Total RNA was extracted using TRIzol reagent (Invitrogen, Carlsbad, CA, USA), subjected to DNase I treatment, and reverse-transcribed using the PrimeScript™ RT kit (TaKaRa Bio Inc., Kusatsu, Shiga, Japan). Transcript abundance was quantified by quantitative real-time PCR (qRT-PCR) using the 2^−ΔΔCt^ method, with the *β-tubulin* gene (*SsTub1*, *SS1G_11660*) serving as the endogenous control. All reactions were performed in three biological replicates and three technical replicates. Primers used for qRT-PCR and gene deletion are listed in Table S1 (Supplementary Materials).

### 2.3. Targeted Gene Disruption and Verification

Targeted gene replacement cassettes for *SsOxy1* and *SsOxy2* were constructed using a split-marker homologous recombination strategy [30] carried out with the pSKH vector. Briefly, the 5′ and 3′ flanking sequences (~1.2 kb each) of the target loci were amplified from genomic DNA of *S. sclerotiorum* strain 1980 and fused to overlapping segments of the hygromycin B phosphotransferase (hph) resistance cassette in the pSKH vector. Protoplast generation (via digestion with 2% lysing enzymes, Sigma-Aldrich, St. Louis, MO, USA) and subsequent PEG-mediated transformation were performed following established protocols [31]. Primary transformants emerging on regeneration medium supplemented with 100 μg/mL hygromycin B were subjected to three successive rounds of hyphal tip purification. Homologous integration was confirmed by diagnostic genomic PCR using primers flanking the target locus and internal hph primers. The complete absence of target gene transcripts was verified by RT-PCR (Figure S1). Primers used for verification are listed in Supplementary Table S1.

We acknowledge that Southern blot analysis was not performed to confirm single-copy integration; however, two independent transformants per knockout displayed consistent phenotypes across all assays, substantially reducing the likelihood that the observed phenotypes are due to ectopic integration events.

### 2.4. Untargeted Metabolomics

Mycelial tissues of the wild-type, Δ*SsOxy1*, and Δ*SsOxy2* strains (three biological replicates each) were harvested after 72 h of growth on cellophane-overlaid PDA. Following liquid nitrogen pulverization, metabolites were extracted using 80% methanol spiked with L-2-chlorophenylalanine as an internal standard. Chromatographic separation was achieved on a Vanquish UHPLC system (Thermo Fisher Scientific, Waltham, MA, USA) equipped with an ACQUITY UPLC HSS T3 column (100 mm × 2.1 mm, 1.8 μm), utilizing a binary mobile phase of 0.1% formic acid in water and acetonitrile. Mass spectrometry data were acquired using an Orbitrap Q Exactive™ HF-X in both positive and negative electrospray ionization modes. Quality control samples (pooled extracts) were injected every ten runs to monitor instrument stability. Peak annotation and metabolite identification were performed by matching *m/z*, retention time, and fragmentation patterns against the mzCloud and mzVault libraries. Identification confidence level: Level 2 (putatively annotated) according to the Metabolomics Standards Initiative, as no authentic standards were used for confirmation. Key metabolites discussed in this study—including hypoxanthine, xanthine, guanine, and GSSG—were identified by high-resolution mass spectrometry with accurate mass matching (<5 ppm), retention time alignment, and MS/MS fragmentation against the mzCloud and mzVault libraries. A total of 2137 metabolites were detected. Differential metabolite accumulation was initially screened using a Variable Importance in Projection (VIP) score ≥ 1 and uncorrected *p* < 0.05 (Student’s *t*-test). To control the false discovery rate in this untargeted metabolomics dataset, Benjamini–Hochberg (BH) multiple testing correction was subsequently applied, and metabolites with FDR (q-value) < 0.15 were considered for biological interpretation. Key metabolites discussed in the main text (GSSG, guanine, hypoxanthine) all exhibited FDR < 0.15, with GSSG (FDR = 0.0297 in Δ*SsOxy1* and 0.0825 in Δ*SsOxy2*) and guanine (FDR = 0.0559 in Δ*SsOxy1* and 0.0557 in Δ*SsOxy2*) showing FDR < 0.06. KEGG pathway enrichment analysis was performed using Fisher’s exact test.

### 2.5. Statistical Analysis

All quantitative assays were performed in biological triplicate unless otherwise stated. Data are presented as mean ± standard deviation (SD). Statistical significance was determined using Student’s *t*-test (two-tailed) for pairwise comparisons or one-way analysis of variance (ANOVA) followed by Tukey’s post hoc test for multiple comparisons, using SPSS 22.0 software. A threshold of *p* < 0.05 was considered statistically significant.

## 3. Results

### 3.1. Structural Attributes and Infection-Induced Transcription of SsOxy1 and SsOxy2

**Figure 1:**
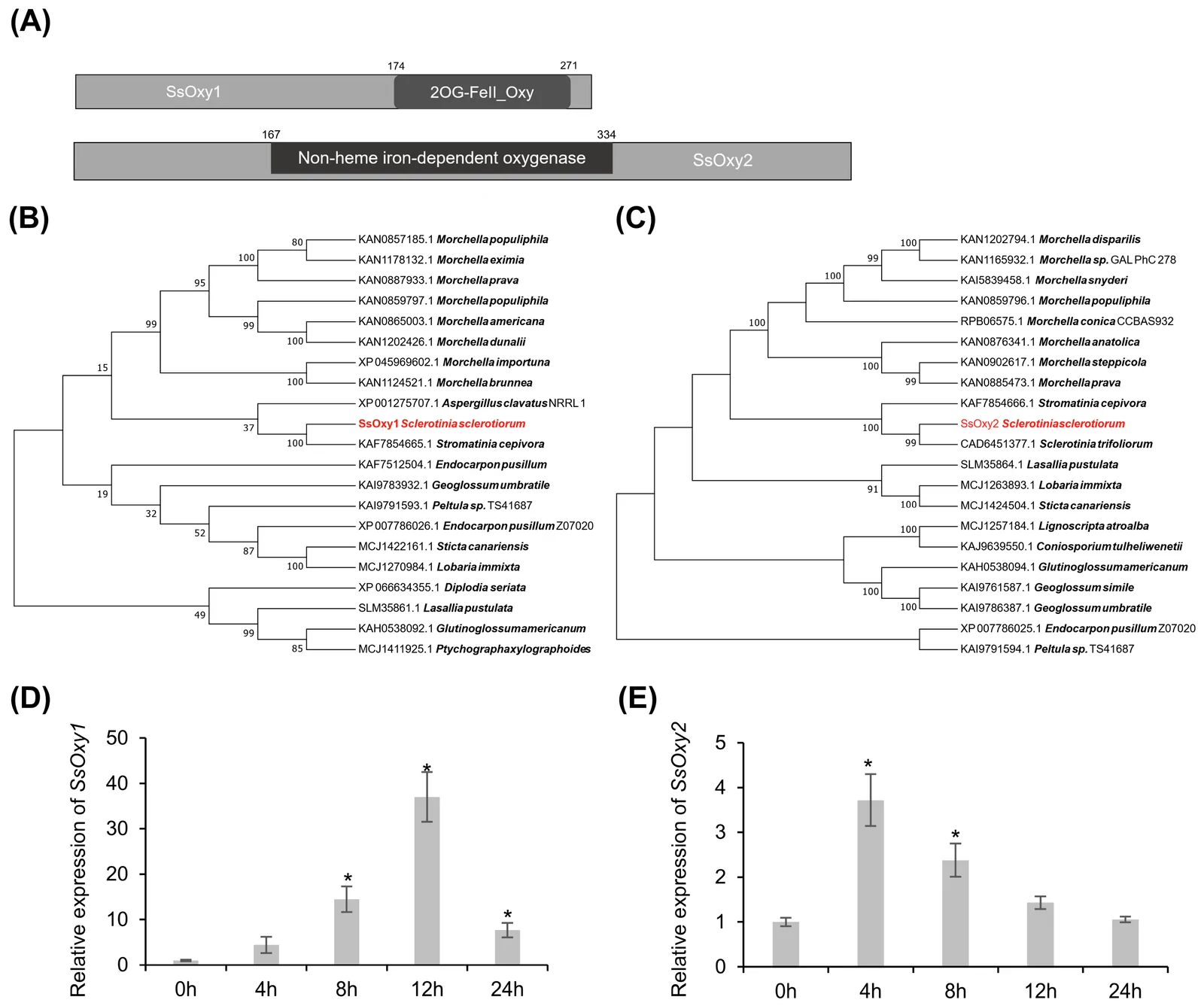
Structural features, phylogenetic relationships, and infection-induced expression dynamics of *SsOxy1* and *SsOxy2*. (**A**) Conserved domain architecture of SsOxy1 and SsOxy2 proteins. SsOxy1 contains a 2-oxoglutarate-dependent oxygenase domain (Pfam 03171, labeled as 2OG-FeII_Oxy), while SsOxy2 harbors a non-heme iron-dependent oxygenase domain (Pfam 14518, labeled as Non-heme_Fe_Oxy). Black boxes indicate the positions of the domains, and numbers correspond to amino acid residues. (**B**) Phylogenetic tree of SsOxy1 based on homologous protein sequences. The tree was constructed using the neighbor-joining method with 1000 bootstrap replicates. Bootstrap support values (top 20 shown) are indicated at the branch nodes. SsOxy1 is highlighted in red. (**C**) Phylogenetic tree of SsOxy2 based on homologous protein sequences. The same method as in (**B**) was used. (**D**) Relative expression of *SsOxy1* during infection of Nicotiana benthamiana leaves. Transcript levels were measured by qRT-PCR, normalized to the endogenous control β-tubulin, and calibrated to the 0 h time point (pre-inoculation, set to 1). Data represent means ± SD from three biological replicates. *SsOxy1* expression peaked at 12 h post-inoculation (approximately 37-fold, * indicates *p* < 0.05, Student’s *t*-test). (**E**) Relative expression of *SsOxy2* during infection of *N. benthamiana* leaves. The same method as in (**D**) was used. *SsOxy2* transcript levels increased rapidly and reached the highest level at 4 h post-inoculation (approximately 3.7-fold, * indicates *p* < 0.05), followed by a gradual decline.

Bioinformatic domain analysis classified *SsOxy1* (*SS1G_07964*) as a 2-oxoglutarate-dependent oxygenase (Pfam 03171) and *SsOxy2* (*SS1G_07963*) as a non-heme iron-dependent oxygenase (Pfam 14518) (Figure 1A). Exhaustive synteny searches (BlastP e-value < 1 × 10^−5^, coverage > 50%) failed to identify direct orthologs in closely related Leotiomycetes such as Botrytis cinerea, suggesting these loci may represent lineage-specific acquisitions in *S. sclerotiorum* (Figure 1B,C). Transcriptional profiling in planta demonstrated a robust, early-stage induction for both genes: *SsOxy2* expression peaked dramatically at 4 hpi (~3.7-fold induction, *p* < 0.01, Figure 1E), whereas *SsOxy1* transcripts reached maximum accumulation at 12 hpi (~37-fold induction, *p* < 0.01, Figure 1D). This staggered transcriptional cascade implies functionally distinct yet coordinated roles during the establishment of host infection.

### 3.2. Divergent Roles in Vegetative Growth and Sclerotial Biogenesis

**Figure 2:**
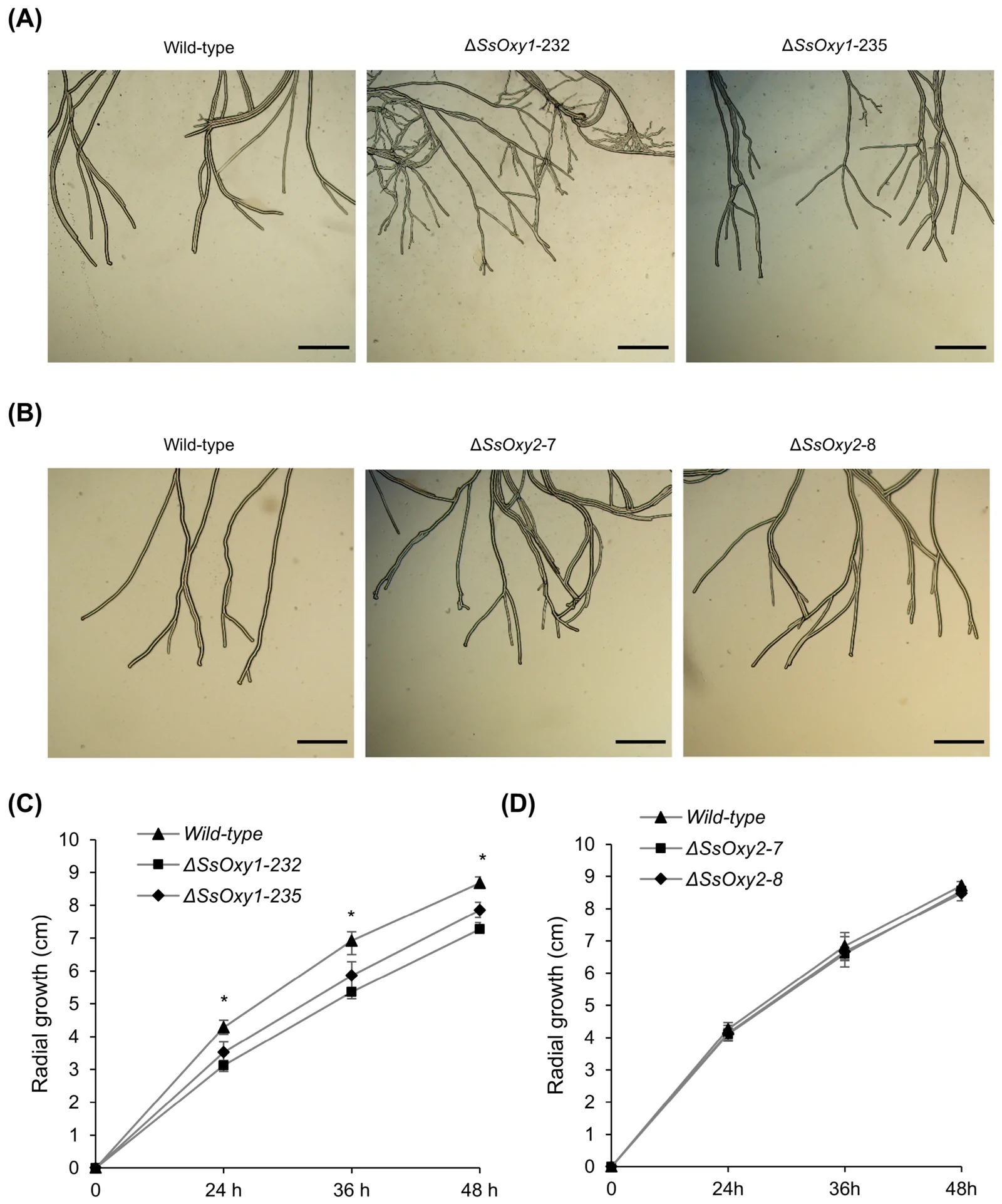
Mycelial morphology and growth rate of Δ*SsOxy1* and Δ*SsOxy2* mutants. (**A**) Microscopic observation of mycelial morphology of the wild-type (WT) and Δ*SsOxy1* strains. Hyphae were grown on PDA plates for 48 h and then examined under a light microscope. Compared with the wild-type, the Δ*SsOxy1* mutant displayed a hyper-branched, compact mycelial architecture, with increased branching frequency and shorter internodal distances. Scale bar = 50 µm. (**B**) Microscopic observation of mycelial morphology of the wild-type (WT) and Δ*SsOxy2* strains. Under the same conditions, no obvious difference in hyphal branching or morphology was observed between Δ*SsOxy2* and the wild-type. Scale bar = 50 µm. (**C**) Time-course of colony radial growth of the wild-type and Δ*SsOxy1* strains. Mycelial plugs (5 mm diameter) were inoculated onto PDA plates, and colony diameters were measured at 0, 24, 36, and 48 h. Data represent means ± SD from three biological replicates. At 24 h post-inoculation, colony diameters were reduced by 27.1% ± 5.0% for Δ*SsOxy1*-232 and 17.8% ± 8.6% for Δ*SsOxy1*-235 compared to the wild-type (* indicates *p* < 0.05, Student’s *t*-test). (**D**) Time course of colony radial growth of the wild-type and Δ*SsOxy2* strains. The same method as in (**C**) was used. No significant difference in radial growth was detected between Δ*SsOxy2* and the wild-type at any time point (*p* > 0.05).

**Figure 3:**
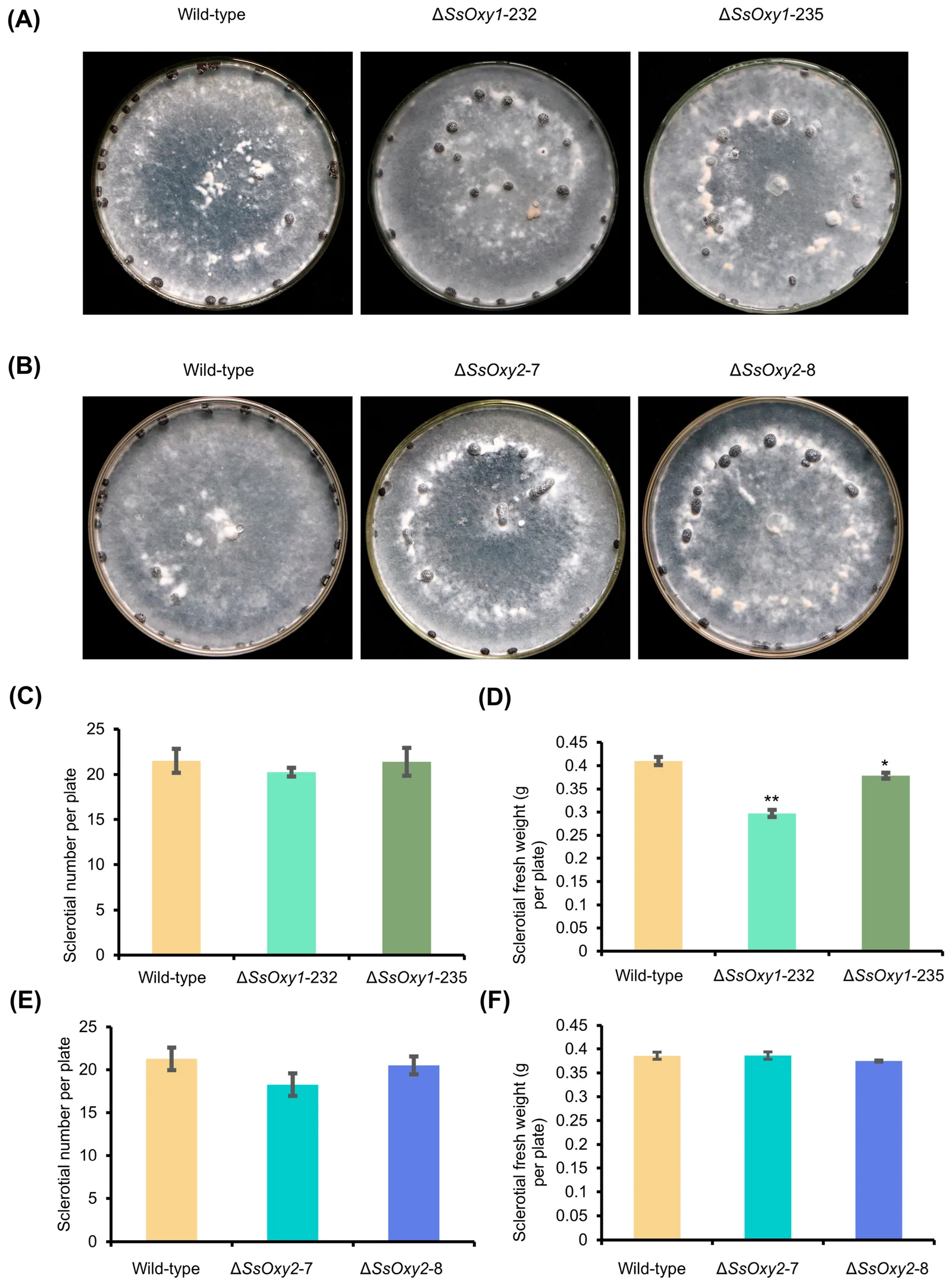
Sclerotial development phenotypes of Δ*SsOxy1* and Δ*SsOxy2* mutants. (**A**) Sclerotial distribution morphology of the wild-type (WT) and Δ*SsOxy1* strains after 20 days of culture on PDA plates. Representative plate photographs show that, unlike the typical concentric ring distribution of the wild-type, Δ*SsOxy1* exhibited an irregular spatial distribution of sclerotia. (**B**) Sclerotial distribution morphology of the wild-type (WT) and Δ*SsOxy2* strains after 20 days of culture on PDA plates. Similar to Δ*SsOxy1*, Δ*SsOxy2* also displayed an irregular spatial distribution of sclerotia, in contrast to the concentric ring pattern of the wild-type. (**C**) Quantification of sclerotial number per plate for the wild-type and Δ*SsOxy1* strains. Data represent means ± SD from three biological replicates. No significant difference in sclerotial number was observed between Δ*SsOxy1* and the wild-type (*p* > 0.05). (**D**) Quantification of sclerotial fresh weight per plate for the wild-type and Δ*SsOxy1* strains. Data represent means ± SD from three biological replicates. Compared with the wild-type (0.410 ± 0.009 g), Δ*SsOxy1*-232 showed 0.297 ± 0.008 g (27.6% ± 1.9% reduction), and Δ*SsOxy1*-235 showed 0.379 ± 0.006 g (7.6% ± 2.5% reduction) (** indicates *p* < 0.01, * indicates *p* < 0.05, Student’s *t*-test). (**E**) Quantification of sclerotial number per plate for the wild-type and Δ*SsOxy2* strains. No significant difference was detected between Δ*SsOxy2* and the wild-type (*p* > 0.05). (**F**) Quantification of sclerotial fresh weight per plate for the wild-type and Δ*SsOxy2* strains. No significant difference was detected between Δ*SsOxy2* and the wild-type (*p* > 0.05).

Precise gene replacement mutants (Δ*SsOxy1* and Δ*SsOxy2*) were generated and validated by PCR and RT-PCR (Figure S1). All phenotypic assays were performed using two independent transformants (Δ*SsOxy1*-232 and Δ*SsOxy1*-235; Δ*SsOxy2*-7 and Δ*SsOxy2*-8), and consistent results were obtained for both transformants. Phenotypic evaluations revealed that the absence of *SsOxy1* imposed a significant penalty on vegetative fitness; colony expansion was restricted by approximately 18% to 27% at 24 h (27.1% ± 5.0% for Δ*SsOxy1*-232 and 17.8% ± 8.6% for Δ*SsOxy1*-235, *p* < 0.05, Figure 2C), accompanied by an atypical hyper-branched, compact mycelial architecture (Figure 2A; qualitative observation; quantitative branching frequency was not measured). Concurrently, Δ*SsOxy1* exhibited a significant reduction in sclerotial fresh weight compared to the wild-type, ranging from approximately 8% to 28% (27.6% ± 1.9% for Δ*SsOxy1*-232 and 7.6% ± 2.5% for Δ*SsOxy1*-235; *p* < 0.01, Figure 3D), and displayed irregular spatial distribution of sclerotia (Figure 3A). Conversely, the Δ*SsOxy2* mutant sustained wild-type radial growth rates (Figure 2D), but also showed a striking disruption in sclerotial spatial patterning (Figure 3B). The irregular sclerotial distribution in both Δ*SsOxy1* and Δ*SsOxy2* indicates a loss of spatial regulation. Furthermore, no significant differences in sclerotial number or fresh weight were detected between Δ*SsOxy2* and the wild-type (Figure 3E,F).

### 3.3. Oxygenase Deficiency Abrogates Infection Cushion Formation and Virulence

**Figure 4:**
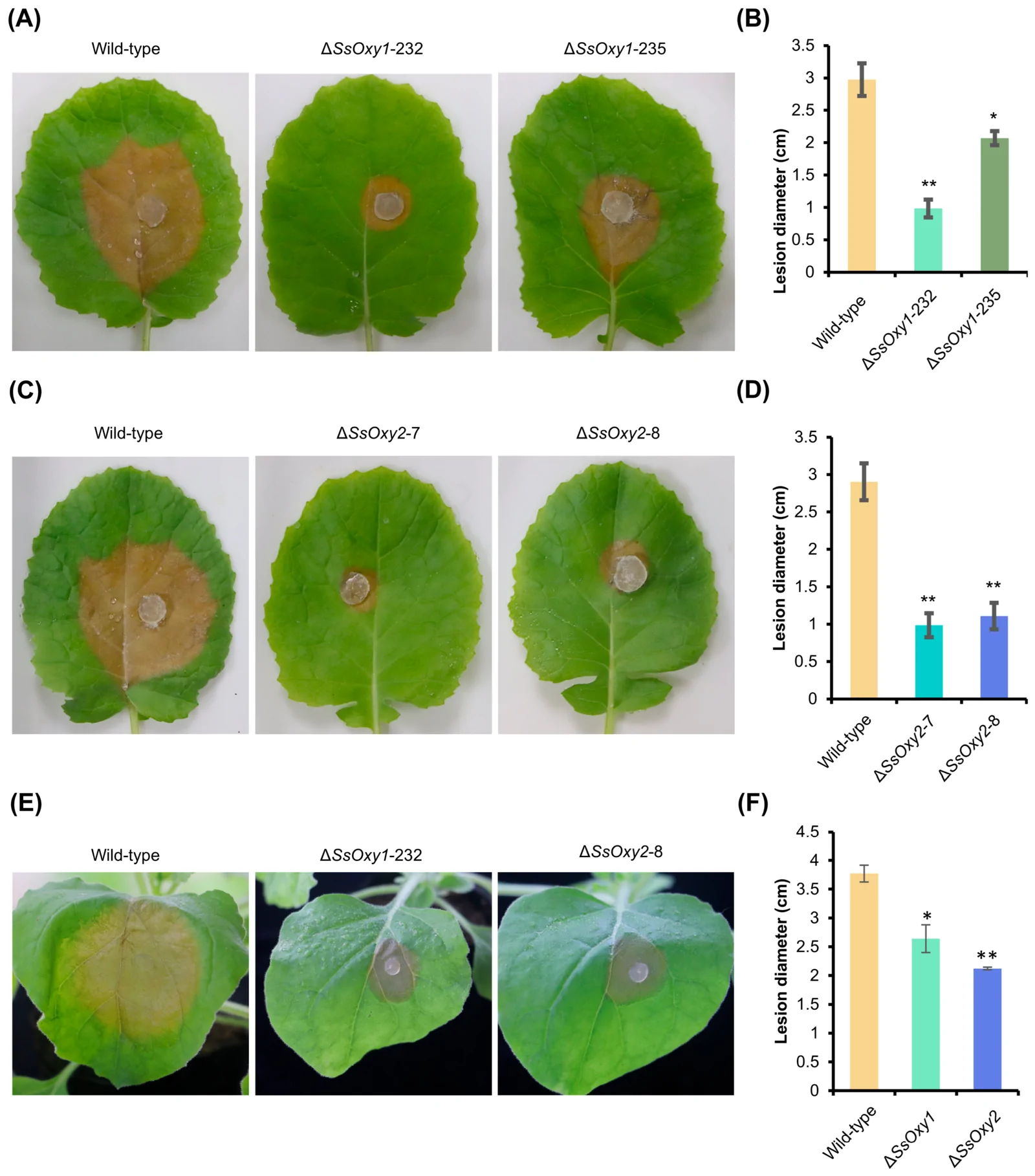
Pathogenicity assays of Δ*SsOxy1* and Δ*SsOxy2* mutants on detached oilseed rape leaves and living *Nicotiana benthamiana* leaves. (**A**) Representative photographs of lesions on detached oilseed rape leaves at 48 h post-inoculation with the wild-type (WT) and Δ*SsOxy1* strains. (**B**) Statistical comparison of lesion diameters on detached oilseed rape leaves. Data represent means ± SD from three biological replicates. The wild-type produced lesions of 2.97 ± 0.25 cm, whereas Δ*SsOxy1*-232 and Δ*SsOxy1*-235 produced lesions of 0.98 ± 0.14 cm (66.9% ± 11.0% reduction) and 2.07 ± 0.11 cm (30.4% ± 3.1% reduction), respectively (** indicates *p* < 0.01, * indicates *p* < 0.05,Student’s *t*-test). (**C**) Representative photographs of lesions on detached oilseed rape leaves at 48 h post-inoculation with the wild-type (WT) and Δ*SsOxy2* strains. (**D**) Statistical comparison of lesion diameters on detached oilseed rape leaves. Data represent means ± SD from three biological replicates. The wild-type produced lesions of 2.90 ± 0.25 cm, whereas Δ*SsOxy2*-7 and Δ*SsOxy2*-8 produced lesions of 0.99 ± 0.16 cm (66.1% ± 14.3% reduction) and 1.11 ± 0.18 cm (61.9% ± 13.0% reduction), respectively (** indicates *p* < 0.01, Student’s *t*-test). (**E**) Representative photographs of lesions on living *Nicotiana benthamiana* leaves at 48 h post-inoculation with the wild-type (WT), Δ*SsOxy1*, and Δ*SsOxy2* strains. Consistent with the results on detached leaves, both mutants also developed markedly smaller lesions on *N. benthamiana*. (**F**) Statistical comparison of lesion diameters on living *N. benthamiana* leaves. Data represent means ± SD from three biological replicates. Both Δ*SsOxy1* and Δ*SsOxy2* showed significantly reduced lesion diameters compared to the wild-type (** indicates *p* < 0.01, * indicates *p* < 0.05, Student’s *t*-test), indicating that deletion of either oxygenase gene severely compromises pathogenicity on both hosts.

**Figure 5:**
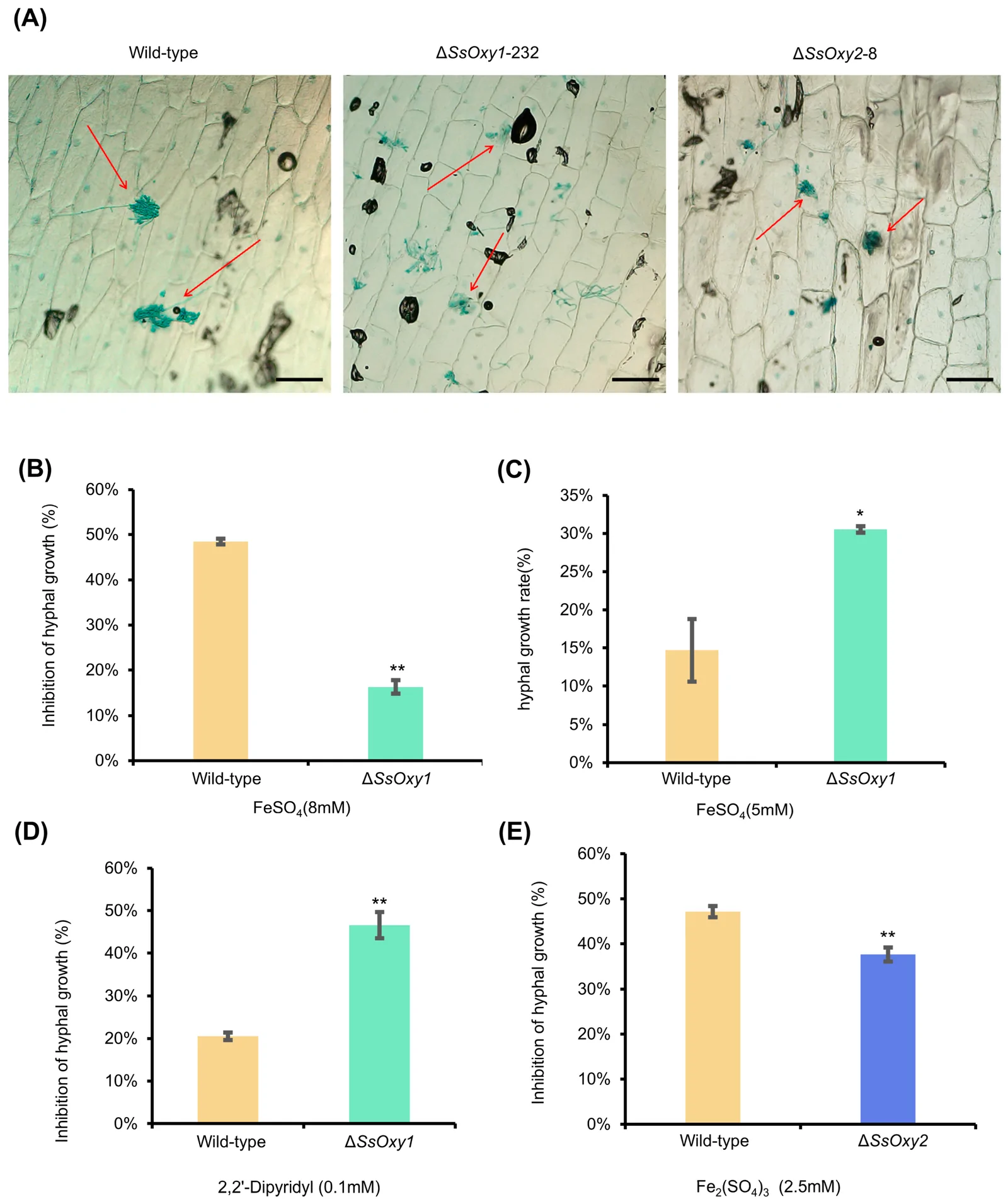
Infection cushion morphology and responses to iron-related stresses of Δ*SsOxy1* and Δ*SsOxy2* mutants. (**A**) Infection cushion formation of the wild-type (WT), Δ*SsOxy1*, and Δ*SsOxy2* strains on onion epidermis at 6 h post-inoculation (lactophenol cotton blue staining). The wild-type developed typical, highly branched infection cushion structures (indicated by arrows), whereas both mutants failed to form intact infection cushions and only sparse hyphae were observed. Scale bar = 200 µm. (**B**) Growth inhibition of the wild-type (WT) and Δ*SsOxy1* strains on PDA medium supplemented with 8 mM FeSO_4_. The relative inhibition rate was calculated as (blank − sample)/blank, with the blank control set as 0% inhibition. Data are means ± SD from three biological replicates. The wild-type showed 48.0% ± 0.6% inhibition, whereas Δ*SsOxy1* showed only 16.0% ± 1.5% inhibition (** indicates *p* < 0.01, Student’s *t*-test), indicating that Δ*SsOxy1* exhibits enhanced tolerance to high concentrations of exogenous Fe^2+^. (**C**) Relative growth rate of the wild-type (WT) and Δ*SsOxy1* strains on PDA medium supplemented with 5 mM FeSO_4_. The relative growth rate was calculated as (sample − blank)/blank, with the blank control set to 0. Data are means ± SD from three biological replicates. Fe^2+^ treatment promoted growth in both strains, with Δ*SsOxy1* showing a significantly higher relative growth rate (0.305 ± 0.004) than the wild-type (0.147 ± 0.041, * indicates *p* < 0.05, Student’s *t*-test). (**D**) Growth inhibition of Δ*SsOxy1* on PDA medium supplemented with 0.1 mM 2,2′-dipyridyl (an iron chelator). Compared with the untreated control, 2,2′-dipyridyl significantly inhibited the growth of Δ*SsOxy1* (** indicates *p* < 0.01, Student’s *t*-test), further confirming that the function of *SsOxy1* is dependent on iron availability. (**E**) Growth inhibition of the wild-type (WT) and Δ*SsOxy2* strains on PDA medium supplemented with 2.5 mM Fe_2_(SO_4_)_3_ (ferric sulfate). The wild-type showed significantly higher inhibition than Δ*SsOxy2* (** indicates *p* < 0.01, Student’s *t*-test), suggesting that *SsOxy2* may also be involved in iron-dependent metabolic pathways.

Standardized pathogenicity assays on detached *B. napus* and living *N. benthamiana* foliage exposed a severe virulence defect in both mutants. By 48 hpi, lesion diameters induced by Δ*SsOxy1* and Δ*SsOxy2* were significantly reduced compared to the wild-type strain (*p* < 0.01, Figure 4B,D,F). Specifically, the wild-type produced lesions of 2.97 ± 0.25 cm, whereas Δ*SsOxy1*-232 and Δ*SsOxy1*-235 produced lesions of 0.98 ± 0.14 cm (66.9% ± 11.0% reduction) and 2.07 ± 0.11 cm (30.4% ± 3.1% reduction), respectively (Figure 4B). Similarly, the wild-type produced lesions of 2.90 ± 0.25 cm, whereas Δ*SsOxy2*-7 and Δ*SsOxy2*-8 produced lesions of 0.99 ± 0.16 cm (66.1% ± 14.3% reduction) and 1.11 ± 0.18 cm (61.9% ± 13.0% reduction), respectively (Figure 4D). To investigate the cytological basis of this attenuation, early infection structures were monitored on onion epidermis. At 6 hpi, the wild-type strain had elaborated dense, highly organized multicellular infection cushions (Figure 5A). In stark contrast, both Δ*SsOxy1* and Δ*SsOxy2* hyphae remained largely undifferentiated, proliferating sparsely across the surface without forming the requisite compound appressoria (Figure 5A). This developmental arrest at the cuticle-penetration interface serves as the primary etiology for their compromised virulence.

### 3.4. Mutants Exhibit Hypersensitivity to Oxidative and Hyperosmotic Stresses

**Figure 6:**
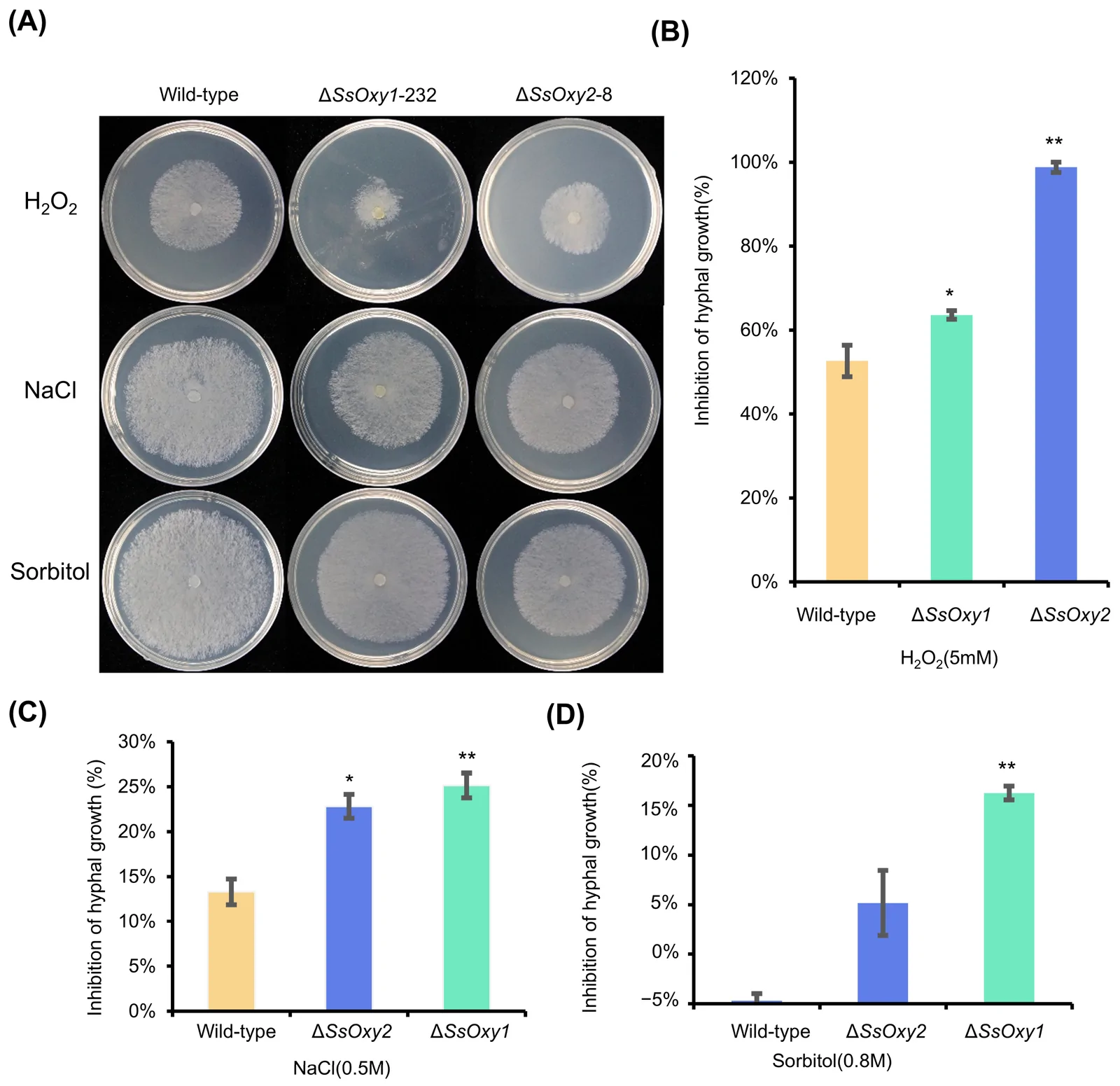
Sensitivity of Δ*SsOxy1* and Δ*SsOxy2* mutants to oxidative and hyperosmotic stresses. (**A**) Representative colony photographs of the wild-type (WT), Δ*SsOxy1*, and Δ*SsOxy2* strains cultured on PDA supplemented with different stress agents for 48 h. Stress conditions included: 5 mM H_2_O_2_ (oxidative stress), 0.5 M NaCl (ionic hyperosmotic stress), and 0.8 M sorbitol (non-ionic hyperosmotic stress). Untreated PDA served as the control. (**B**) Growth inhibition rates of the wild-type (WT), Δ*SsOxy1*, and Δ*SsOxy2* strains on PDA containing 5 mM H_2_O_2_ (relative to their respective untreated controls set as 0% inhibition). Data represent means ± SD from three biological replicates. Compared with the wild-type, both mutants exhibited significantly higher inhibition rates (** indicates *p* < 0.01, * indicates *p* < 0.05, Student’s *t*-test), indicating increased sensitivity to oxidative stress. (**C**) Growth inhibition rates of the wild-type (WT), Δ*SsOxy1*, and Δ*SsOxy2* strains on PDA containing 0.5 M NaCl. Method as in (**B**). Both mutants showed significantly higher inhibition rates than the wild-type (** indicates *p* < 0.01, * indicates *p* < 0.05, Student’s *t*-test), indicating increased sensitivity to ionic hyperosmotic stress. (**D**) Growth inhibition rates of the wild-type (WT), Δ*SsOxy1*, and Δ*SsOxy2* strains on PDA containing 0.8 M sorbitol. Method as in (**B**). Compared with the wild-type, Δ*SsOxy1* showed a significantly higher inhibition rate (** indicates *p* < 0.01, Student’s *t*-test), while Δ*SsOxy2* exhibited a trend toward higher inhibition that did not reach statistical significance (*p* > 0.05), suggesting differential responses of the two mutants to non-ionic hyperosmotic stress.

Exposure to 5 mM H_2_O_2_ elicited pronounced growth inhibition in Δ*SsOxy1* (63.6% ± 1.0%, *p* < 0.05) and Δ*SsOxy2* (98.8% ± 1.2%, *p* < 0.01), a stark deviation from the mild inhibition observed in the wild-type (52.6% ± 3.7%, Figure 6B). Similar hypersensitivity was recorded under hyperosmotic stress (0.5 M NaCl), where mutant growth was suppressed by approximately 25.1% ± 1.38% (*p* < 0.01) and 22.8% ± 1.33% (*p* < 0.05), respectively, compared to 13.3% ± 1.44%for the control (*p* < 0.01, Figure 6C). Under non-ionic hyperosmotic stress (0.8 M sorbitol), Δ*SsOxy1* exhibited significantly higher growth inhibition (16.25% ± 0.69%) compared to the wild-type (−4.71% ± 0.73%, *p* < 0.01), whereas Δ*SsOxy2* (5.18% ± 3.29%) showed no significant difference from the wild-type (*p* > 0.05, Figure 6D). To assess the iron dependence of *SsOxy1* and *SsOxy2* function, we performed complementary experiments with different endpoints (Figure 5B–E). Figure 5B shows the relative inhibition rate in medium supplemented with 8 mM FeSO_4_, calculated as (blank − sample)/blank. Under this condition, the wild-type was severely inhibited (48.0% ± 0.6%), whereas Δ*SsOxy1* showed markedly lower inhibition (16.0% ± 1.5%, *p* < 0.01), indicating enhanced tolerance to high concentrations of exogenous Fe^2+^. Figure 5C shows the relative growth rate on medium supplemented with 5 mM FeSO_4_, calculated as (sample − blank)/blank. Interestingly, Fe^2+^ treatment promoted growth in both strains, with Δ*SsOxy1* showing a significantly higher relative growth rate (0.305 ± 0.004) than the wild-type (0.147 ± 0.041, *p* < 0.05). Figure 5D shows the inhibition rate on medium containing the iron chelator 2,2′-dipyridyl (0.1 mM), which significantly inhibited Δ*SsOxy1* growth (*p* < 0.01), further confirming that *SsOxy1* function is iron-dependent. Similarly, Δ*SsOxy2* exhibited significantly lower growth inhibition than the wild-type when cultured on medium supplemented with 2.5 mM Fe_2_(SO_4_)_3_ (*p* < 0.01, Figure 5E), suggesting that *SsOxy2* may also be involved in iron-dependent metabolic pathways. These biochemical complementation data substantiate the functional requirement of iron for *SsOxy1* activity and broadly implicate both oxygenases in the pathogen’s adaptive response to oxidative and osmotic fluctuations.

### 3.5. Metabolomic Profiling Uncovers Reprogramming of Purine and Glutathione Metabolism and Additional Metabolic Disturbances

Untargeted LC-MS/MS metabolomics captured 2137 distinct analytes across samples. Comparative profiling identified 100 differential metabolites in Δ*SsOxy1* and 103 in Δ*SsOxy2* (VIP ≥ 1, *p* < 0.05). Strikingly, the mutants shared a core set of 63 differentially accumulated metabolites (38 upregulated, 25 downregulated; Figure S2). KEGG pathway enrichment analysis of these shared metabolites (Table S2) revealed highly significant enrichments in purine metabolism (*p* = 6.2 × 10^−5^), glutathione metabolism (*p* = 1.8 × 10^−3^), and biosynthesis of secondary metabolites (*p* = 3.5 × 10^−3^).

Quantitative analysis of key differential metabolites is summarized in Table 1. Crucially, the purine intermediates—hypoxanthine, xanthine, and guanine—were significantly depleted in both mutants. In Δ*SsOxy1*, hypoxanthine, xanthine, and guanine were reduced by approximately 4.6-fold (log_2_FC = −2.193), 8.1-fold (log_2_FC = −3.010), and 3.7-fold (log_2_FC = −1.872), respectively (all *p* < 0.05; Table 1). In Δ*SsOxy2*, the reductions were approximately 4.9-fold (log_2_FC = −2.285), 6.1-fold (log_2_FC = −2.610), and 2.5-fold (log_2_FC = −1.309), respectively (all *p* < 0.01; Table 1). Concurrently, oxidized glutathione (GSSG) was significantly elevated in both mutants: 3.0-fold in Δ*SsOxy1* (log_2_FC = +1.580, FDR = 0.0297) and 5.7-fold in Δ*SsOxy2* (log_2_FC = +2.512, FDR = 0.0825), indicating a disrupted GSH/GSSG redox balance (Table 1). Moreover, two short peptides, Asp-Glu and Gly-Phe, were significantly downregulated in both mutants (1.3- to 1.8-fold, *p* < 0.05).

Beyond these core pathways, the mutants exhibited widespread reprogramming of lipids, carbohydrates, and phenolic compounds (Table S3). In Δ*SsOxy1*, several diacylglycerols (e.g., DAG (4:0/4:0)) and lysophospholipids (e.g., BMP (5:0/17:1)) were significantly downregulated (log_2_FC < −1.5), suggesting potential impairments in membrane lipid signaling and expansion. Carbohydrate metabolites, including maltose and raffinose, were decreased by 1.5- to 2.5-fold in both mutants, which may compromise osmoprotection and carbon reserves. Interestingly, multiple plant-derived phenolic acids (ferulic acid, syringic acid) and isoflavonoids were markedly elevated (up to 8-fold) in the mutants. Notably, ferulic acid and the isoflavonoid daidzein were specifically and dramatically elevated in Δ*SsOxy2* (log_2_FC = +6.65 and +7.95, respectively), whereas Δ*SsOxy1* showed a broader depletion of lipids and carbohydrates (Table S3). These observations may reflect mutant-specific responses to xenobiotic stress or altered secondary metabolism, although the underlying mechanisms remain to be determined.

**Figure 7:**
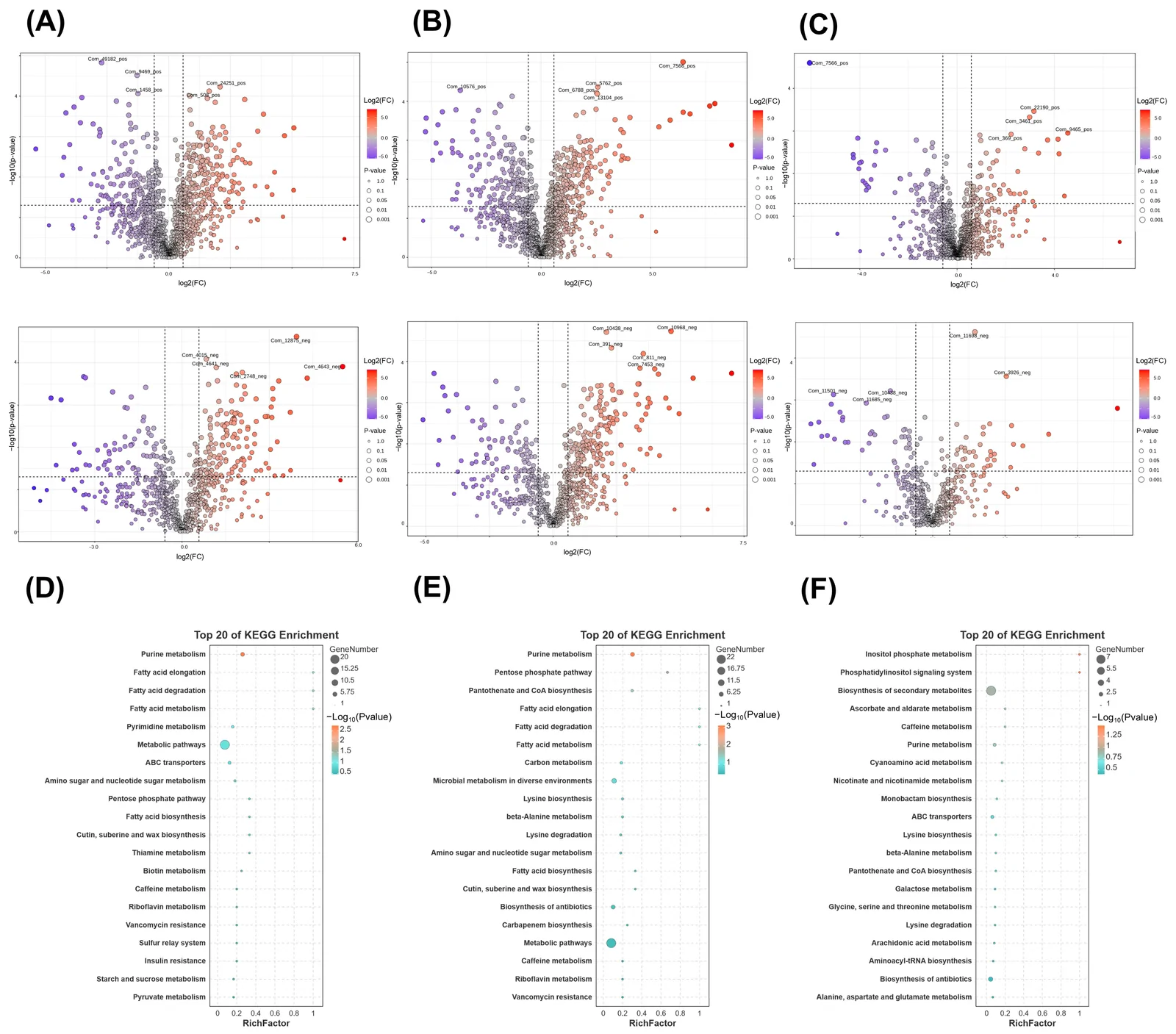
Differential metabolite analysis and pathway enrichment of Δ*SsOxy1* and Δ*SsOxy2* mutants. (**A**) Volcano plot of differential metabolites between Δ*SsOxy1* and the wild-type (WT). Upper panel: positive ion mode; lower panel: negative ion mode. The x-axis represents the fold change (log_2_ fold change), and the y-axis represents the significance level (−log_10_ *p*-value). Red dots indicate significantly upregulated metabolites, blue dots indicate significantly downregulated metabolites (VIP ≥ 1, *p* < 0.05), and gray dots indicate non-significant metabolites. (**B**) Volcano plot of differential metabolites between Δ*SsOxy2* and the wild-type (WT). Upper panel: positive ion mode; lower panel: negative ion mode. The same method as in (**A**) was used. (**C**) Volcano plot of differential metabolites between Δ*SsOxy1* and Δ*SsOxy2*. Upper panel: positive ion mode; lower panel: negative ion mode. The same method as in (**A**) was used. (**D**) Bubble chart of KEGG pathway enrichment for the top 20 most significantly differential metabolites between Δ*SsOxy1* and the wild-type (ranked by significance). Bubble size represents the number of differential metabolites in the pathway, and bubble color represents the enrichment significance (*p*-value; darker color indicates higher significance). The x-axis is the rich factor, and the y-axis is the pathway name. (**E**) Bubble chart of KEGG pathway enrichment for the top 20 most significantly differential metabolites between Δ*SsOxy2* and the wild-type. The same method as in (**D**) was used. (**F**) Bubble chart of KEGG pathway enrichment for the top 20 most significantly differential metabolites between Δ*SsOxy1* and Δ*SsOxy2*. The same method as in (**D**) was used.

To further examine the metabolic differences between the two mutants, we compared Δ*SsOxy1* directly with Δ*SsOxy2*. Figure 7F shows the KEGG pathway enrichment of differential metabolites between the two mutants, which included pathways related to purine metabolism, glutathione metabolism, and amino acid metabolism. The most significantly altered metabolites between the two mutants are listed in Table 2 (top 10 by VIP) and Table S4 (biologically relevant metabolites). Notably, Δ*SsOxy1* accumulated higher levels of GSSG (log_2_FC = −0.932, *p* = 0.0125) and 3′-AMP(log_2_FC = +2.608 relative to Δ*SsOxy2*, *p* = 0.00096), whereas Δ*SsOxy2* exhibited a trend toward greater depletion of hypoxanthine(log_2_FC = +0.444, *p* = 0.063). These subtle differences may reflect distinct kinetic or regulatory properties of the two oxygenases.

This profound metabolomic reprogramming aligns with the observed physiological deficits in energy-intensive infection cushion formation and oxidative stress tolerance.

## 4. Discussion

Oxygenases constitute a diverse and evolutionarily refined class of oxidoreductases that are indispensable for the structural diversification of fungal secondary metabolites. These metabolic products act as critical chemical mediators during pathogen–plant interactions, governing morphogenetic transitions, host subversion, and environmental resilience [32,33]. In the current study, genetic ablation of two functionally cryptic, infection-induced oxygenase genes—*SsOxy1* and *SsOxy2*—unmasked their essential, pleiotropic contributions to the life cycle and pathogenic competence of *S. sclerotiorum*.

The most severe phenotypic consequence of deleting either *SsOxy1* or *SsOxy2* was the near-total abrogation of infection cushion development, which directly precipitated a massive reduction in virulence. The elaboration of infection cushions is a heavily energy-demanding morphogenetic program, requiring rapid cytoskeletal reorganization, localized turgor generation, and the massive exocytosis of effector proteins and cell wall-degrading enzymes [34]. To elucidate the biochemical root of this developmental arrest, untargeted metabolomics proved invaluable. The drastic depletion of purine intermediates (hypoxanthine, xanthine, guanine) in both mutants suggests a systemic collapse in nucleotide homeostasis. Purine metabolism is the foundational pathway for generating cellular energy currencies (ATP, GTP). The depletion of these purine intermediates is consistent with a perturbation of nucleotide homeostasis, which may limit the availability of ATP and GTP required for the intense metabolic demands of appressorial differentiation. Direct quantification of ATP and GTP pools via targeted metabolomics will be required to test this hypothesis. This paradigm is strongly corroborated by research in the rice blast fungus Magnaporthe oryzae, where targeted disruptions in de novo purine biosynthesis absolutely abolish in planta proliferation and pathogenicity [35]. It is highly plausible that *SsOxy1* and *SsOxy2* interface, either directly or via metabolic crosstalk, with purine salvage pathways, thereby sustaining the energetic threshold required for cuticle breaching. However, the current metabolomic data only demonstrate correlation, not causation; future complementation experiments with exogenous purine intermediates are needed to establish causality.

Beyond energetic deficits, both mutants displayed heightened vulnerability to exogenous H_2_O_2_, a phenotype elegantly mirrored by their disrupted glutathione pools. During the incipient stages of infection, plants deploy a rapid oxidative burst as a primary antimicrobial defense. Necrotrophic success relies on the pathogen’s ability to neutralize this reactive oxygen species barrage, traditionally achieved through the secretion of oxalic acid and the mobilization of intracellular antioxidants [36]. The observed accumulation of oxidized glutathione (GSSG) and the concomitant drop in the GSH/GSSG ratio definitively diagnose a state of chronic intracellular oxidative stress in the mutants. This redox imbalance allows for two non-mutually exclusive mechanistic hypotheses: (i) *SsOxy1* and *SsOxy2* may function as direct reactive oxygen species scavengers by utilizing H_2_O_2_ during substrate oxidation; (ii) more likely, they are critical enzymes required for the biosynthesis of an antioxidant secondary metabolite that indirectly supports redox balance. Distinguishing between these possibilities will require in vitro enzyme activity assays and the identification of the putative secondary metabolite.

Notably, despite encoding distinct functional domains, Δ*SsOxy1* and Δ*SsOxy2* yielded remarkably convergent phenotypes across morphological, pathogenic, and metabolomic metrics. This phenotypic parity strongly implies that these genes do not act in isolation but rather operate within a shared biochemical cascade.

Direct metabolomic comparison between Δ*SsOxy1* and Δ*SsOxy2* revealed distinctive metabolic signatures beyond their shared purine and glutathione perturbations. Notably, 3′-AMP—a specific marker of RNA degradation—was dramatically elevated exclusively in Δ*SsOxy1* (log_2_FC = +2.608 relative to Δ*SsOxy2*, *p* = 0.00096; Table 2), suggesting that loss of *SsOxy1* may be associated with global RNA instability. This observation offers a plausible metabolic explanation for the compromised vegetative growth (Figure 2C) and the more pronounced depletion of guanine and xanthine pools observed specifically in Δ*SsOxy1*. In contrast, Δ*SsOxy2* exhibited a greater accumulation of GSSG (5.7-fold vs. 3.0-fold in Δ*SsOxy1*), indicating a more severe redox imbalance, yet its radial growth remained unaffected (Figure 2D), implying the engagement of compensatory mechanisms that remain to be identified. These differential signatures suggest that *SsOxy1* and *SsOxy2*, while functionally linked in core pathways (purine metabolism and redox homeostasis), contribute to distinct aspects of cellular homeostasis—RNA stability for *SsOxy1* and redox adaptation for *SsOxy2*.

Inspection of the *S. sclerotiorum* genome reveals that *SsOxy1* and *SsOxy2* are contiguous and flanked by genes encoding a transporter and a flavoprotein. This specific topological arrangement is consistent with the genomic architecture of a putative fungal biosynthetic gene cluster [25]. However, we acknowledge that direct evidence for co-transcription of the entire putative cluster, or the identification of a shared biosynthetic product, is currently lacking. Comprehensive in silico surveys have previously mapped numerous transcriptionally co-regulated, silent biosynthetic gene clusters within the sub-telomeric regions of the *S. sclerotiorum* genome [25,37]. The metabolomic depletion of specific dipeptides (Asp-Glu, Gly-Phe) in both mutants provides a compelling clue regarding the nature of this putative cluster. Nonribosomal peptide synthetases and cyclic dipeptide synthases frequently recruit juxtaposed oxygenases to perform iterative oxidative tailoring—such as cross-linking, epoxidation, or hydroxylation—on the nascent peptide scaffold [37]. We hypothesize that *SsOxy1* and *SsOxy2* constitute the core modifying machinery of an uncharacterized nonribosomal peptide synthetase-driven biosynthetic gene cluster. Loss of either oxygenase would abort maturation of the final peptide product, triggering feedback inhibition that downregulates upstream precursors and disrupts global cellular homeostasis, thereby manifesting as identical developmental and pathogenic defects. We acknowledge that direct evidence for co-transcription of the putative cluster genes is currently lacking, and the term “cooperatively” rather than “synergistically” more accurately reflects the current level of proof.

In summary, this study reveals for the first time the critical roles of the *S. sclerotiorum* oxygenases *SsOxy1* and *SsOxy2* in infection cushion formation, maintenance of pathogenicity, and regulation of redox homeostasis. They likely regulate pathogen development and pathogenesis in multiple dimensions by affecting purine metabolism-mediated energy supply, participating in reactive oxygen species scavenging or antioxidant production, and in a coordinated manner completing the biosynthesis of a peptide-derived secondary metabolite. We acknowledge several limitations in this study. First, genetic complementation strains were not generated; while consistent phenotypes across multiple independent transformants support the specificity of the gene-deletion phenotypes, reintroducing wild-type copies of *SsOxy1* and *SsOxy2* into the respective mutants would provide definitive confirmation of causality. Second, Southern blot analysis was not performed to confirm single-copy integration; however, the split-marker system is well-established for targeted deletion in *S. sclerotiorum* [30,31], and multiple independent transformants with consistent phenotypes reduce concerns about ectopic integration. Third, the key metabolomic identifications (hypoxanthine, xanthine, guanine, and GSSG) were made at Level 2 (putatively annotated) based on high-resolution mass spectrometry (< 5 ppm), retention time alignment, and MS/MS fragmentation matching against public spectral libraries (mzCloud and mzVault), but without authentication using genuine standards; targeted metabolomics with authentic standards would further strengthen these assignments in future studies. Despite these limitations, the convergence of independent lines of evidence—phenotypic characterization, stress response assays, infection cushion microscopy, and untargeted metabolomics—supports the robustness of our core conclusions.

## 5. Conclusions

This study provides the first systematic functional characterization of the paired oxygenase genes *SsOxy1* and *SsOxy2* in *Sclerotinia sclerotiorum*. Genetic analyses demonstrated that *SsOxy1* governs vegetative vigor and sclerotial biomass, while *SsOxy2* strictly regulates the spatial patterning of sclerotia. Crucially, the absence of either enzyme halts the differentiation of infection cushions, severely attenuating virulence and rendering the pathogen hypersensitive to oxidative stress. Metabolomic integration identified a collapse in purine metabolism and glutathione-mediated redox buffering as the biochemical mechanisms underlying these phenotypic failures. Genomic context and metabolic profiling converge to suggest that *SsOxy1* and *SsOxy2* may act in a coordinated manner as tailoring enzymes within a novel secondary metabolite gene cluster. Ultimately, these findings illuminate the intricate regulatory networks linking secondary metabolism, energy allocation, and morphogenetic transitions in *S. sclerotiorum*, providing a foundation for future exploration of oxygenases as potential fungicidal targets.

## Figures and Tables

**Table 1:** Key differential metabolites in purine metabolism, glutathione metabolism, and dipeptides in Δ*SsOxy1* and Δ*SsOxy2* mutants.

Metabolite	Comparison	log_2_FC	*p*-Value	FDR	VIP	Regulation	Pathway
Hypoxanthine	Δ*SsOxy1*/WT	−2.193	0.0246	0.1485	6.665	Down	Purine metabolism
Hypoxanthine	Δ*SsOxy2*/WT	−2.285	0.0230	0.1418	7.312	Down	Purine metabolism
Xanthine	Δ*SsOxy1*/WT	−3.010	0.0016	0.0553	1.472	Down	Purine metabolism
Xanthine	Δ*SsOxy2*/WT	−2.610	0.0035	0.0983	1.273	Down	Purine metabolism
Guanine	Δ*SsOxy1*/WT	−1.872	0.00047	0.0559	5.580	Down	Purine metabolism
Guanine	Δ*SsOxy2*/WT	−1.309	0.00033	0.0557	5.237	Down	Purine metabolism
L-Glutathione oxidized (GSSG)	Δ*SsOxy1*/WT	+1.580	6.69 × 10^−5^	0.0297	4.936	Up	Glutathione metabolism
L-Glutathione oxidized (GSSG)	Δ*SsOxy2*/WT	+2.512	0.00178	0.0825	7.811	Up	Glutathione metabolism
Asp-Glu	Δ*SsOxy1*/WT	−0.512	0.0379	0.1684	1.049	Down	Peptide metabolism
Asp-Glu	Δ*SsOxy2*/WT	−0.821	0.00619	0.0994	1.297	Down	Peptide metabolism
Gly-Phe	Δ*SsOxy1*/WT	−0.524	0.00092	0.0559	1.335	Down	Peptide metabolism
Gly-Phe	Δ*SsOxy2*/WT	−0.420	0.0104	0.1126	1.207	Down	Peptide metabolism

Note: Data are from untargeted LC-MS/MS metabolomics. Comparisons are Δ*SsOxy1* vs wild-type (WT) and Δ*SsOxy2* vs WT. log_2_FC: fold change (positive = up, negative = down). VIP: variable importance in projection (OPLS-DA). *p*-values from Student’s *t*-test (uncorrected). FDR: false discovery rate (Benjamini–Hochberg corrected). All listed metabolites satisfy VIP ≥ 1 and *p* < 0.05.

**Table 2:** Differential metabolites between Δ*SsOxy1* and Δ*SsOxy2* (top 10 by VIP).

Metabolite	log_2_FC (ΔOxy1/ΔOxy2)	*p*-Value	VIP	Trend (Relative to ΔOxy2)
L-Glutathione oxidized (GSSG)	−0.932	0.0125	9.59	Lower in ΔOxy1
Guanine	−0.563	0.0474	3.68	Lower in ΔOxy1
Arecoline	+2.229	0.0067	4.37	Higher in ΔOxy1
Rauwolscine	+1.930	0.0022	4.97	Higher in ΔOxy1
3′-Adenosine monophosphate (3′-AMP)	+2.608	0.00096	4.73	Higher in ΔOxy1
Cytidine-5′-monophosphate	+2.163	0.0418	2.57	Higher in ΔOxy1
PG (3:0/20:4)	+1.932	0.0061	1.82	Higher in ΔOxy1
PA (2:0/13:0)	+1.963	0.00997	1.43	Higher in ΔOxy1
Adenosine 5′-monophosphate	+1.009	0.0191	1.21	Higher in ΔOxy1
7-Methylxanthine	+0.866	0.0128	1.05	Higher in ΔOxy1

Note: Comparison is Δ*SsOxy1* vs. Δ*SsOxy2*. Positive log_2_FC indicates higher abundance in Δ*SsOxy1*; negative indicates higher abundance in Δ*SsOxy2*. Sorted by VIP descending. All metabolites satisfy VIP ≥ 1 and *p* < 0.05.

## Data Availability

The original contributions presented in this study are included in the article/Supplementary Materials (Tables S1–S5, Figures S1 and S2). The complete lists of differential metabolites for all comparisons in positive and negative ion modes are provided in Table S5. Further inquiries can be directed to the corresponding authors.

## Supplementary Materials

The following supporting information can be downloaded at: https://www.mdpi.com/article/10.3390/jof12090688/s1. Figure S1. PCR and RT-PCR validation of Δ*SsOxy1* and Δ*SsOxy2* knockout mutants. (A) Diagnostic genomic PCR confirming homologous integration of the *SsOxy1* knockout construct. Primer pair: KO*SsOxy1*fp/KO*SsOxy1*rp (target gene-specific; expected band in wild-type only). M: DNA marker; WT: wild-type; Δ*SsOxy1*: Δ*SsOxy1* mutant. (B) RT-PCR confirming the absence of *SsOxy1* transcripts in the Δ*SsOxy1* mutant. Total RNA was reverse-transcribed and amplified with gene-specific primers. Primer pair: q*SsOxy1*-F/q*SsOxy1*-R. (C) β-Tubulin gene (*SsTub1*) served as the loading control for the Δ*SsOxy1* mutant. Primer pair: q*SsTub1*-F/q*SsTub1*-R. (D) Diagnostic genomic PCR confirming homologous integration of the *SsOxy2* knockout construct. Primer pair: KO*SsOxy2*fp/KO*SsOxy2*rp (target gene-specific; expected band in wild-type only). M: DNA marker; WT: wild-type; Δ*SsOxy2*: Δ*SsOxy2* mutant. (E) RT-PCR confirming the absence of *SsOxy2* transcripts in the Δ*SsOxy2* mutant. Total RNA was reverse-transcribed and amplified with gene-specific primers. Primer pair: q*SsOxy2*-F/q*SsOxy2*-R. (F) *β-Tubulin* gene (*SsTub1*) served as the loading control for the Δ*SsOxy2* mutant. Primer pair: q*SsTub1*-F/q*SsTub1*-R. Figure S2. Quality control of untargeted metabolomics data and heatmaps of metabolic profiles. (A) PCA plot of quality control (QC) samples in positive ion mode. The tight clustering of QC samples indicates stable instrument performance and good data reproducibility throughout the analytical run. (B) PCA plot of QC samples in negative ion mode. The tight clustering of QC samples further confirms data stability and reliability in negative ion mode. (C) Clustered heatmap of all metabolites detected in positive ion mode for the wild-type (WT), Δ*SsOxy1*, and Δ*SsOxy2* strains. Each column represents a biological replicate, and each row represents a metabolite. Color intensity indicates relative metabolite abundance (red: high; blue: low). The heatmap reveals distinct metabolic profiles among the three genotypes. (D) Clustered heatmap of all metabolites detected in negative ion mode for the wild-type (WT), Δ*SsOxy1*, and Δ*SsOxy2* strains. The same method as in (C) was used. The results further confirm metabolic reprogramming between the mutants and the wild-type. Table S1. Primers used in this study. Note: List of primers for gene deletion (split-marker) and qRT-PCR. Tm: annealing temperature. Table S2. KEGG pathway enrichment analysis of shared differential metabolites (top 10). Note: Based on the 63 shared differential metabolites between Δ*SsOxy1* and Δ*SsOxy2*. Rich factor = number of differential metabolites in pathway/total metabolites in pathway. *p*-values from Fisher’s exact test. FDR-adjusted values for individual metabolites are provided in Table 1 and Table S5. Table S3. Representative differential metabolites in lipid, carbohydrate, and phenolic classes. Note: Selected from significant changes (VIP ≥ 1, *p* < 0.05) in Δ*SsOxy1* or Δ*SsOxy2* vs wild-type. Only metabolites with potential biological relevance are shown. n.d. = not detected as a differential metabolite. FDR values are provided where available. Table S4. Biologically relevant differential metabolites between Δ*SsOxy1* and Δ*SsOxy2*. Note: Based on Δ*SsOxy1* vs Δ*SsOxy2* comparison (VIP ≥ 1, *p* < 0.05). Grouped by biological implication (oxidative stress, purine turnover, signaling, detoxification). FDR values are provided. Table S5. Complete lists of differential metabolites for all comparisons in positive and negative ion modes. Note: The file contains six sheets: *Oxy1*_pos, *Oxy1*_neg, *Oxy2*_pos, *Oxy2*_neg, *Oxy1*_vs_*Oxy2*_pos, and *Oxy1*_vs_*Oxy2*_neg. Each sheet includes metabolite name, log2FC, *p*-value, FDR, VIP, and molecular formula. All metabolites satisfy VIP ≥ 1 and *p* < 0.05.

## Abbreviations

The following abbreviations are used in this manuscript:

2-OG	2-Oxoglutarate
2,2′-Bipy	2,2′-Bipyridyl
3′-AMP	3′-Adenosine monophosphate
ANOVA	Analysis of variance
ATP	Adenosine triphosphate
BGC	Biosynthetic gene cluster
*B. napus*	*Brassica napus*
*B. cinerea*	*Botrytis cinerea*
CDD	Conserved Domain Database
cDNA	Complementary DNA
CRISPR	Clustered regularly interspaced short palindromic repeats
CWDEs	Cell wall-degrading enzymes
DAG	Diacylglycerol
DNA	Deoxyribonucleic acid
FDR	False discovery rate
Fe^2+^	Ferrous ion
Fe_2_(SO_4_)_3_	Ferric sulfate
FeSO_4_	Ferrous sulfate
GSH	Reduced glutathione
GSSG	Oxidized glutathione
GTP	Guanosine triphosphate
h	Hour(s)
hpi	Hours post-inoculation
hph	Hygromycin B phosphotransferase
H_2_O_2_	Hydrogen peroxide
ISR	Induced systemic resistance
KEGG	Kyoto Encyclopedia of Genes and Genomes
LC-MS/MS	Liquid chromatography-tandem mass spectrometry
MEGA	Molecular Evolutionary Genetics Analysis
M. oryzae	Magnaporthe oryzae
NaCl	Sodium chloride
NCBI	National Center for Biotechnology Information
*N. benthamiana*	*Nicotiana benthamiana*
NRPS	Nonribosomal peptide synthetase
OA	Oxalic acid
OPLS-DA	Orthogonal partial least squares discriminant analysis
PCR	Polymerase chain reaction
PDA	Potato dextrose agar
PEG	Polyethylene glycol
PR1	Pathogenesis-related protein 1
QC	Quality control
qRT-PCR	Quantitative real-time PCR
RNA	Ribonucleic acid
ROS	Reactive oxygen species
SA	Salicylic acid
SAR	Systemic acquired resistance
SD	Standard deviation
SEM	Standard error of the mean
*S. sclerotiorum*	*Sclerotinia sclerotiorum*
SOD	Superoxide dismutase
UDP	Uridine diphosphate
UHPLC	Ultra-high-performance liquid chromatography
VIP	Variable importance in projection
WT	Wild-type
