# Supplementary material for: Functional Characterization of Two Adjacent Oxygenase Genes, SsOxy1 and SsOxy2, Reveals Their Critical Roles in Sclerotial Development, Stress Tolerance, and Pathogenicity of Sclerotinia sclerotiorum
Source: J Fungi (Basel). 2026 Sep 13;12(9):688. doi: 10.3390/jof12090688 (PMC13608281; doi:10.3390/jof12090688)
Supplement: Supplementary file 1 [file jof-12-00688-s001.zip › Supplementary.pdf]

Figure S1

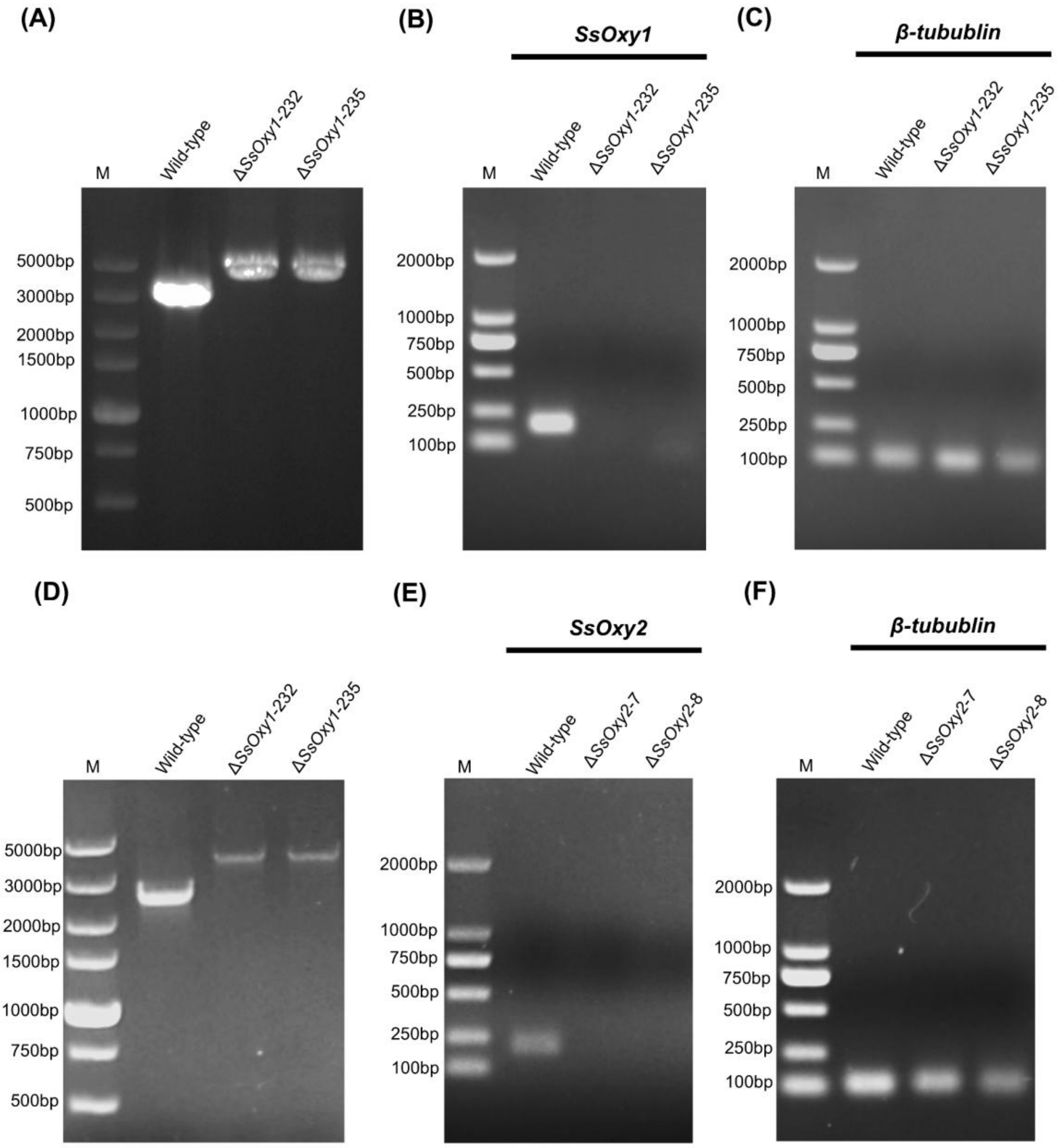

Figure S1. PCR and RT-PCR validation of Δ*SsOxy1* and Δ*SsOxy2* knockout mutants..**(A)** Diagnostic genomic PCR confirming homologous integration of the *SsOxy1* knockout construct. Primer pair: KO*SsOxy1*fp/KO*SsOxy1*rp (target gene-specific; expected band in wild-type only). M: DNA marker; WT: wild-type; Δ*SsOxy1*: Δ*SsOxy1* mutant. **(B)** RT-PCR confirming the absence of *SsOxy1* transcripts in the Δ*SsOxy1* mutant. Total RNA was reverse-transcribed and amplified with gene-specific primers. Primer pair: q*SsOxy1*-F/q*SsOxy1*-R. **(C)** *β-Tubulin* gene (*SsTub1*) served as the loading control for the Δ*SsOxy1* mutant. Primer pair: q*SsTub1*-F/q*SsTub1*-R. **(D)** Diagnostic genomic PCR confirming homologous integration of the *SsOxy2* knockout construct.

Primer pair: KO*SsOxy2*fp/KO*SsOxy2*rp (target gene-specific; expected band in wild-type only). M: DNA marker; WT: wild-type; Δ*SsOxy2*: Δ*SsOxy2* mutant. **(E)** RT-PCR confirming the absence of *SsOxy2* transcripts in the Δ*SsOxy2* mutant. Total RNA was reverse-transcribed and amplified with gene-specific primers. Primer pair: q*SsOxy2*-F/q*SsOxy2*-R. **(F)** *β-Tubulin* gene (*SsTub1*) served as the loading control for the Δ*SsOxy2* mutant. Primer pair: q*SsTub1*-F/q*SsTub1*-R.

Figure S2

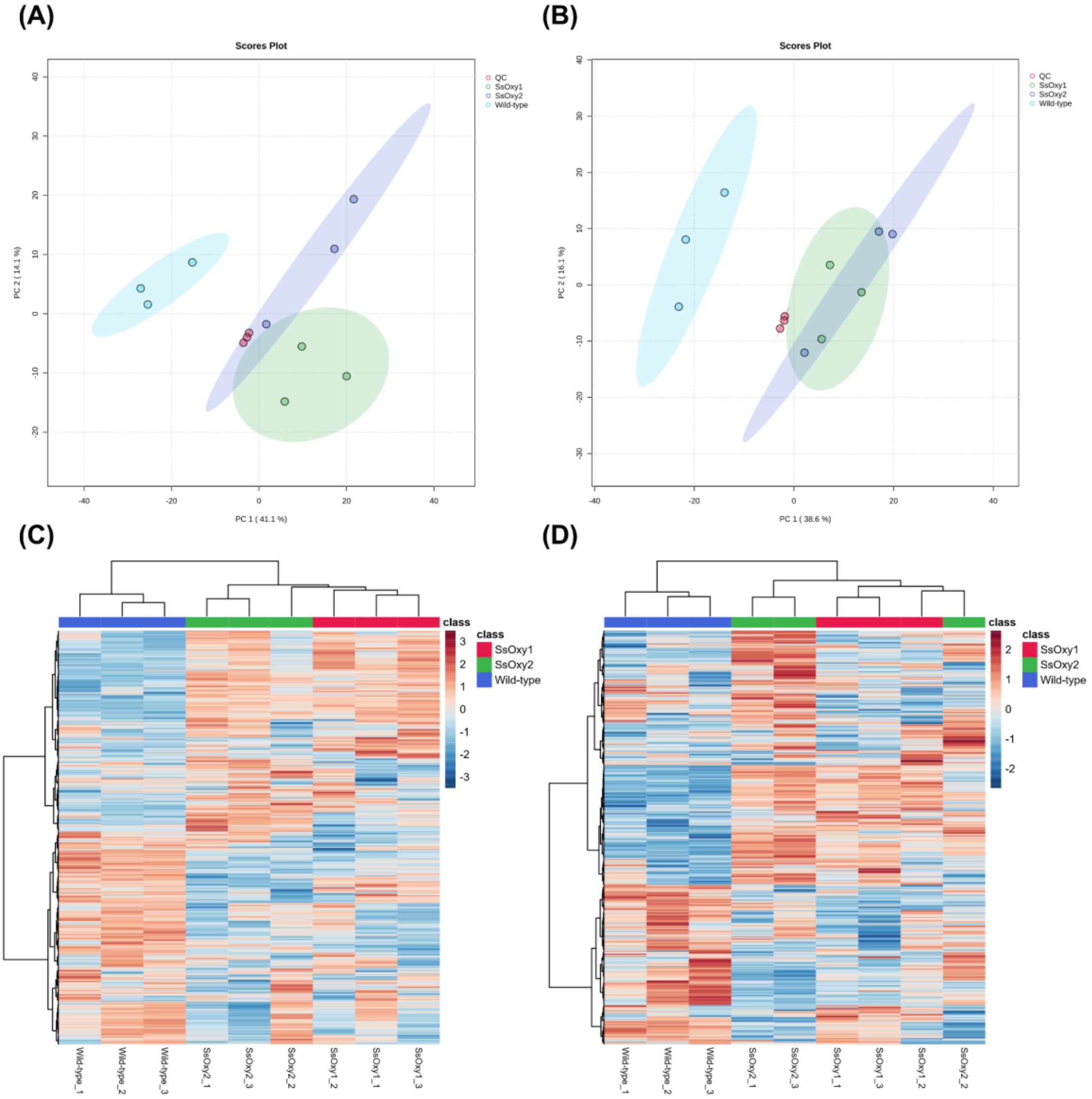

Figure S2. Quality control of untargeted metabolomics data and heatmaps of metabolic profiles. **(A)** Principal component analysis (PCA) plot of quality control (QC) samples in positive ion mode. The tight clustering of QC samples indicates stable instrument performance and good data reproducibility throughout the analytical run. **(B)** PCA plot of QC samples in negative ion mode. The tight clustering of QC samples further confirms data stability and reliability in negative ion mode. **(C)** Clustered heatmap of all metabolites detected in positive ion mode for the wild-type (WT), Δ*SsOxy*1, and Δ*SsOxy2* strains. Each column represents a biological replicate, and each row represents a metabolite. Color

intensity indicates relative metabolite abundance (red: high; blue: low). The heatmap reveals distinct metabolic profiles among the three genotypes. **(D)** Clustered heatmap of all metabolites detected in negative ion mode for the wild-type (WT), Δ*SsOxy1*, and Δ*SsOxy2* strains. The same method as in (C) was used. The results further confirm metabolic reprogramming between the mutants and the wild-type.

Table S1

Table S1. Primers used in this study

Primer name	Sequence (5′→3′)	Tm (°C)
SsOxy1-UNotI	ATAAGAATGCGGCCGCTGCCCCACCTTCATCACTTC	60
SsOxy1-USalI	CGCGTCGACGCTCATCCCGGAGATCAAGC	60
SsOxy1-DSmaI	TGCCCCGGGCGCTCGATTAGCTGAGATGAGA	60
SsOxy1-DKpnI	CGCGGTACCGTATTTACAGGGCACCCCGT	60
SsOxy2-UNotI	ATAAGAATGCGGCCGCACATGCAATACGACGTTATGTGC	60
SsOxy2-USalI	CGCGTCGACCTTCAATCAACTCCAAGCCTGA	60
SsOxy2-DSpeI	CGGACTAGTCCAGATCACTGTTGAGGAGATCAA	60
SsOxy2-DKpnI	CGGGGTACCTCCAAACTAACCTAGAAGAGGAGTG	60
*SsOxy1*U	TGCCTCACAACACTCTGCAAA	60
*SsOxy2*U	TCATGCACACAACTCCCA	60
*SsOxy1*D	CTTAATCGCCTTCTCGGGCT	60
*SsOxy2*D	TCGCCTCTTAATCTCGGGCT	60
HY	AAATTGCCGTCAACCAAGCTC	60
YG	TTTCAGCTTCGATGTAGGAGG	60
KO*SsOxy1*fp	GCTGAACTGTAGAGATTGACTTGCA	60
KO*SsOxy1*rp	TGGTCACTTTCTCACAATTCCTCA	60
KO*SsOxy2*fp	GTAAGCGTCCTCTCTTATATGACGC	60
KO*SsOxy2*rp	ACCTGCTTCAATTTCATACTCCAA	60
q*SsOxy1*-F	ACGAACCCCTCAAACACAAGA	60
q*SsOxy1*-R	TGCAACGAGTCGGCAACAT	60
q*SsOxy2*-F	TCGCTCCCAAACCACTCTTC	60
q*SsOxy2*-R	ATCGTAATTCATCCATGCCG	60
q*SsTub1*-F	TTGGATTTGCTCCTTTGACCAG	60
q*SsTub1*-R	AGCGGCCATCATGTTCTTAGG	60

* Note: List of primers for gene deletion (split-marker) and qRT-PCR. Tm: annealing temperature.

Table S2

Table S2. KEGG pathway enrichment analysis of shared differential metabolites (top 10)

Pathway	Differentially abundant metabolites	Rich factor	P-value

Purine metabolism	6	0.065	6.2×10^{-5}
Glutathione metabolism	4	0.143	1.8×10^{-3}
Biosynthesis of secondary metabolites	8	0.026	3.5×10^{-3}
ABC transporters	3	0.068	1.2×10^{-2}
Amino acid metabolism	3	0.025	2.8×10^{-2}
Pentose phosphate pathway	2	0.071	3.9×10^{-2}
Fatty acid metabolism	1	0.028	4.7×10^{-2}
Microbial metabolism in diverse environments	5	0.032	5.1×10^{-2}
Starch and sucrose metabolism	1	0.024	6.3×10^{-2}
Pyrimidine metabolism	1	0.021	7.8×10^{-2}

* Note: Based on the 63 shared differential metabolites between Δ*SsOxy1* and Δ*SsOxy2*. Rich factor = number of differential metabolites in pathway / total metabolites in pathway. P-values from Fisher's exact test. FDR-adjusted values for individual metabolites are provided in Table 1 and Table S5. This table reports pathway-level enrichment P-values based on Fisher's exact test.

Table S3

Table S3. Representative differential metabolites in lipid, carbohydrate, and phenolic classes

Metabolite	Class	ΔOxy1 vs WT (log_2FC)	ΔOxy2 vs WT (log_2FC)	P-value (Δ*Oxy1*)	P-value (Δ*Oxy2*)	FDR (Δ*Oxy1* / WT)	FDR (Δ*Oxy2* / WT)	Biological implication
DAG (4:0/4:0)	Diacylglycerol	-2.088	-1.472	0.0189	0.00482	0.1399	0.0969	Membrane lipid signaling and expansion
BMP (5:0/17:1)	Lysophospholipid	-4.544	-1.440	0.0131	1.03×10^{-5}	0.1157	0.0069	Membrane stability
Maltose	Disaccharide	-0.866	-1.513	0.0263	0.0179	0.1499	0.1273	Carbon reserve, osmoprotection
Raffinose	Trisaccharide	-1.824	-1.513	0.0232	0.0336	0.1529	—	Osmoregulation
Ferulic acid	Phenolic acid	+6.648	+3.677	—	0.00568	n.d.	0.1143	Antioxidant, detoxification
Syringic acid	Phenolic acid	+3.027	+3.740	0.0131	0.00662	0.1157	0.1170	Antioxidant
Daidzein	Isoflavonoid	n.d.	+0.447	—	0.0317	n.d.	0.3925	Xenobiotic metabolism
6''-O-Acetylglycitin	Isoflavonoid	+2.269	+2.341	0.0119	0.0366	0.1120	0.1741	Xenobiotic metabolism

* Note: Selected from significant changes (VIP ≥ 1, P < 0.05) in Δ*SsOxy1* or Δ*SsOxy2* vs wild-type. Only metabolites with potential biological relevance are shown. n.d. = not detected as a differential metabolite (VIP < 1 or P > 0.05). Ferulic acid and daidzein were specifically elevated in Δ*SsOxy2** and were not detected as differential metabolites in Δ*SsOxy1*.

Table S4

Table S4. Biologically relevant differential metabolites between Δ*SsOxy1* and Δ*SsOxy2*

Metabolite	Class	log_2FC (Δ*Oxy1*/Δ*Oxy2*)	P-value	FDR	Trend (relative to Δ*Oxy2*)	Biological implication
Oxidized glutathione (GSSG)	Glutathione	-0.932	0.0125	0.5353	Higher in Δ*Oxy1*	More severe oxidative stress in Δ*Oxy1*
3′-Adenosine monophosphate (3′-AMP)	Purine nucleotide	+2.608	0.00096	0.6407	Higher in Δ*Oxy1*	Altered purine turnover
Hypoxanthine	Purine base	+0.444	0.063	0.4495	Lower in Δ*Oxy2* (trend)	Δ*Oxy2* consumes more hypoxanthine
Guanine	Purine base	-0.563	0.0474	0.3925	Lower in Δ*Oxy1*	Opposite pattern to hypoxanthine
Arecoline	Alkaloid	+2.229	0.0067	0.3925	Higher in Δ*Oxy1*	Possibly detoxification
DAG (4:0/4:0)	Diacylglycerol	-0.616	0.019	0.0969	Lower in Δ*Oxy1*	Lipid signaling difference

* Note: Based on Δ*Oxy1* vs Δ*Oxy2* comparison (VIP ≥ 1, P < 0.05). Grouped by biological implication (oxidative stress, purine turnover, signaling, detoxification). FDR values for metabolites identified in K3-vs-K4 comparison (direct comparison between Δ*SsOxy1* and Δ*SsOxy2*) are provided. Due to the smaller differences between the two mutant backgrounds compared to WT-vs-mutant comparisons, FDR values are inherently less stringent in this direct comparison.
